# Immunoglobulin Heavy Chain High-Throughput Sequencing in Pediatric B-Precursor Acute Lymphoblastic Leukemia: Is the Clonality of the Disease at Diagnosis Related to Its Prognosis?

**DOI:** 10.3389/fped.2022.874771

**Published:** 2022-05-30

**Authors:** Gabriel Levy, Michal Kicinski, Jona Van der Straeten, Anne Uyttebroeck, Alina Ferster, Barbara De Moerloose, Marie-Francoise Dresse, Christophe Chantrain, Bénédicte Brichard, Marleen Bakkus

**Affiliations:** ^1^de Duve Institute, Université Catholique de Louvain, Brussels, Belgium; ^2^Ludwig Institute for Cancer Research, Brussels, Belgium; ^3^Department of Pediatric Oncology and Hematology, Cliniques Universitaires Saint-Luc, Université Catholique de Louvain, Brussels, Belgium; ^4^European Organization for Research and Treatment of Cancer (EORTC) Headquarters, Brussels, Belgium; ^5^Molecular Hematology Laboratory, Vrije Universiteit Brussel, Universitair Ziekenhuis Brussel, Brussels, Belgium; ^6^Department of Pediatric Hemato-Oncology, UZ Leuven, Leuven, Belgium; ^7^Department of Pediatric Hematology-Oncology, Children’s University Hospital Queen Fabiola, Université Libre de Bruxelles (ULB), Brussels, Belgium; ^8^Department of Pediatric Hematology-Oncology and Stem Cell Transplantation, Ghent University Hospital, Ghent, Belgium; ^9^Department of Pediatrics, Centre Hospitalier Régional (CHR) de la Citadelle, Liège, Belgium; ^10^Division of Pediatric Hematology-Oncology, Centre Hospitalier Chrétien (CHC) MontLégia, Liège, Belgium

**Keywords:** minimal residual disease (MRD), BCP-ALL, clonal evolution analysis, prognostic factors, high-throughput sequencing (HTS)

## Abstract

High-throughput sequencing (HTS) of the immunoglobulin heavy chain (*IgH*) locus is a recent very efficient technique to monitor minimal residual disease of B-cell precursor acute lymphoblastic leukemia (BCP-ALL). It also reveals the sequences of clonal rearrangements, therefore, the multiclonal structure, of BCP-ALL. In this study, we performed *IgH* HTS on the diagnostic bone marrow of 105 children treated between 2004 and 2008 in Belgium for BCP-ALL in the European Organization for Research and Treatment of Cancer (EORTC)-58951 clinical trial. Patients were included irrespectively of their outcome. We described the patterns of clonal complexity at diagnosis and investigated its association with patients’ characteristics. Two indicators of clonal complexity were used, namely, the number of foster clones, described as clones with similar D-N_2_-J rearrangements but other V-rearrangement and N_1_-joining, and the maximum across all foster clones of the number of evolved clones from one foster clone. The maximum number of evolved clones was significantly higher in patients with *t*(12;21)/*ETV6:RUNX1*. A lower number of foster clones was associated with a higher risk group after prephase and *t*(12;21)/*ETV6:RUNX1* genetic type. This study observes that clonal complexity as accessed by *IgH* HTS is linked to prognostic factors in childhood BCP-ALL, suggesting that it may be a useful diagnostic tool for BCP-ALL status and prognosis.

## Key Points

1. *IgH* high-throughput sequencing allows new insights into the clonal architecture of BCP-ALL.
2. A higher number of evolved clones at diagnosis of BCP-ALL was associated with the presence of *t*(12;21)/*ETV6:RUNX1*.
3. Patients with a higher number of foster clones were patients in the better prognosis group.

## Introduction

B-cell precursor acute lymphoblastic leukemia (BCP-ALL) is the most common pediatric neoplasm (1, 2). It is a clonal genetic heterogeneous disease generally thought to arise from the malignant transformation and expansion of a single lymphoid progenitor at various stages of development (3–5). The precise pathogenetic events leading to the development of ALL are still unknown, but evidence supports the hypothesis of driver mutations followed by secondary events, that can occur in subclones of the original leukemic cell following different evolution patterns (5, 6).

**FIGURE 1:**
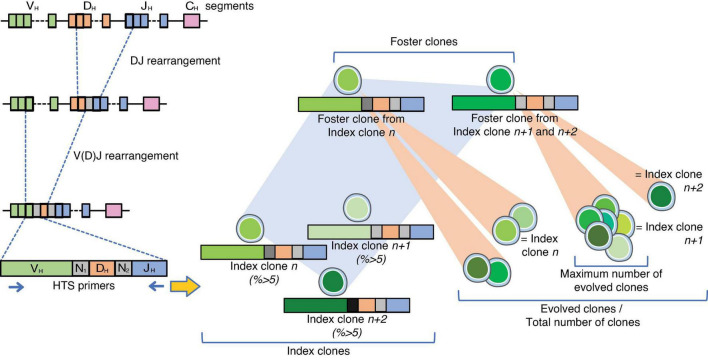
Design of the experiments. On the left panel, the steps for the V(D)J somatic recombination of the *IgH* locus are represented. Diversity is enabled through a random combination of one of many variables (*V*), diversity (*D*), and joining (*J*) gene segments although the major contributor to the diversity of the immunoglobulin repertoire is the variable truncation of the recombined gene segments in synergy with the addition of non-templated (N) nucleotides, the so-called N regions within the rearrangements (8). Index clones were designated as clones representing ≥5% of the individual clonotypes with the same V(D)J rearrangement. Analysis of the V(D)J-sequence allowed identification of related foster clones among index clones. Foster clones were defined as clones with similar D-N_2_-J rearrangements, but other V-rearrangements and N_1_-joining, regardless of their percentage. Evolved clones were clones related to the index and foster clones by sharing the same or partly the same D-J stem, regardless of their frequency. The total number of clones per patient was the sum of evolved clones, and the maximum number of evolved clones was the highest number of evolved clones across all foster clones.

Early in B-cell development, somatic recombinations at the immunoglobulin heavy chain (*IgH*) locus give rise to unique rearrangements resulting from the random coupling between one of the many possible variable (*V*_*H*_), diversity (*D*), and joining (*J*_*H*_) genes [V(D)J recombination or combinatorial diversity], as well as imprecise joining of gene segments and the addition of nucleotides to the DNA sequence at splice sites (N-diversity or junctional diversity) (Figure 1) (7, 8). Identical *IgH* rearrangements, which are a unique signature to B-cells, reflect the clonal nature of a population, and reversely, the clonality of B-cell populations can be assessed by *IgH* analysis. By extension, *IgH* rearrangements constitute clonotypic markers and allow high-resolution tracking of the architecture and clonal dynamic of BCP-ALL cells (3, 9).

In fact, *IgH* studies in paired diagnosis, treatment follow-up, and relapse samples revealed that leukemic cells maintain ongoing *IgH* changes alongside the disease (10, 11), in particular *V*_*H*_ replacement. These changes give insight into the continuous evolution of the BCP-ALL structure with a given number of leukemic subclones that are present at diagnosis or can appear during treatment and possibly reemerge at relapse alongside a dominant clone (3, 9, 10).

Minimal residual disease (MRD), reflecting treatment efficiency, is considered to be the strongest prognostic factor in both children and adult ALL, independently of traditional prognostic factors, such as age, blast count at diagnosis, immunophenotype, or genetic abnormalities (12–15). The term MRD describes a level of disease that is undetectable by conventional cytomorphology and is not accompanied by any clinical symptom. Current methods to monitor MRD in ALL include multicolor flow cytometric (MFC) detection of aberrant immunophenotypes, allele-specific oligonucleotide RQ-PCR (ASO-PCR) amplification of immunoglobulin (Ig), and T-cell receptor (TCR) genes and/or real-time quantitative polymerase chain reaction (RQ-PCR) of fusion transcripts (16–19). Although MFC, ASO-, or RQ-PCR methods are used in a clinical setting and have proven reliable to reach high sensitivity, they all have their own limitations. Among those, MFC can lead to a false-negative finding if antigen expression changes over the course of the disease, fusion transcript RQ-PCR is applicable only in patients with target fusion genes and faces limited standardization, and Ig/TCR ASO-PCR is time-consuming as it needs optimization of patient-specific reagents and assays, which themselves are prone to false-negative results following clonal evolution of the disease (16, 18).

Next-generation sequencing (NGS)-based methods, also called deep- or high-throughput sequencing (HTS) methods, can be used to monitor MRD by detailed sequencing of the V(D)J junctions (16, 17, 20–23).

Next-generation sequencing methods have the advantage to allow quick access to the full IgH repertoire of an individual, without the necessity to develop personalized assays, and have proven to give a more complete insight into the leukemic population than conventional ASO-PCR at each time-point of the MRD monitoring. In comparison to current methods of MRD measurement that had limited to no capacity to monitor the evolution of leukemic subclones during treatment, they thus allow follow-up of subclones and/or identification of new emerging clones throughout the evolution of the disease (3, 21, 24).

Therefore, these methods are sources of great promises, and their major limitation in children resides currently in the need for standardized bioinformatics methods to interpret thoroughly the results of cohort studies to validate this approach (16, 23).

Along with their development for monitoring MRD, HTS methods have also shed light on mechanisms associated with leukemic clonal evolution that were previously underappreciated and have allowed studying leukemic cell evolution according to their niche. For example, Bartram et al. were able to demonstrate that central nervous system (CNS) and bone marrow (BM) clones are the same alongside the evolution of the disease and that BM infiltration would be present at some level, even in apparently isolated CNS relapse (25).

Comprehension of the molecular pathways involved in V(D)J recombinations is still being investigated, as is their relation with genetic instability and potential oncogenicity (26). Papaemmanuil et al. demonstrated in 2014 the relation between leukemogenesis in *t*(12;21)/*ETV6:RUNX1* positive childhood BCP-ALL leukemia and the RAG recombinase activity, an endonuclease required for V(D)J recombination (27). Furthermore, RAG-mediated aberrant recombinations might also be involved in the evolution of *t*(9;22)/BCR-ABL positive BCP-ALL (28, 29).

Nevertheless, to date, there is less understanding of the association between the clonal architecture of the disease at diagnosis and its clinical presentation or prognosis. In this study, we investigated the association between the number of leukemic clones, as defined by clonal *IgH* rearrangements, the number of evolved clones, as defined by the same D-J stem, and the characteristics of patients and known prognostic factors of BCP-ALL at diagnosis.

## Materials and Methods

### Samples

The samples of this study originated from patients registered in the European Organization for Research and Treatment of Cancer (EORTC)-58951 study for the treatment of ALL or lymphoblastic non-Hodgkin’s lymphoma in children between one and 18 years (Supplementary Figure 1) (30, 31).

A subgroup of Belgian patients treated for BCP-ALL between 2004 and 2008 was systematically reviewed for *IgH* recombinations. Diagnostic BM samples with a clonal *IgH* rearrangement and sufficient leftover material were retained for the study. The patients were selected with no knowledge of their outcomes. A total of 105 samples that fulfilled inclusion criteria were used for NGS analysis.

Mononuclear cells (MNCs) had been separated by Lymphoprep™ density gradient centrifugation (Elitech – Cat. No. AX-1114547) from BM aspirates at diagnosis, and genomic DNA was extracted using the QIAamp^®^ DNA Blood Mini Kit (QIAGEN^®^). The DNA was stored at −20°C for later use.

### V(D)J Sequence Analysis

Next-generation sequencing for *IgH* was performed using the LymphoTrack^®^ IGH FR1-MiSeq^®^ kit (Invivoscribe^®^, Cat. No. 91210039). A maximum of 50 ng diagnostic DNA was amplified and sequenced. The primers used for sequencing targeted the framework region 1 (FR1) and the *J*_*H*_ region. The library was sequenced with the MiSeq^®^ device (Illumina^®^, 2 × 250 cycles) at a final concentration of 14 pM and 1% PhiX. A minimum of 20.000 reads had to be obtained for each sample. Sequencing results were aligned with IMGT/V-QUEST (7).

### Definitions Used for the Classification of the Clones

Index clones: the frequency of every clonotype in each sample was determined by calculating the number of sequencing reads for each clonotype divided by the total number of sequencing reads in the sample. In line with former studies (3, 32), an index clone was designated as a clone representing ≥5% of the individual clonotypes (Figure 1).

Foster clones: analysis of the V(D)J-sequence allowed identification of related foster clones among index clones. Foster clones were defined as clones with similar D-N_2_-J rearrangements, but other V-rearrangement and N_1_-joining, regardless of their percentage. The foster clones were manually sorted by searching all the sequences with the same D-N_2_-J region; a minimum of 1/2 of the D-region was used. In case of no D-region, a minimum of 1/2 of the N-region was used. A foster clone indicator was given to each foster clone.

Evolved clones: clones related to the foster clone by sharing the same or partly the same D-J stem, regardless of their frequency. The total number of evolved clones per patient was defined as the sum of clones across all index clones, where the number of clones per index clone was equal to the number of clones evolved from the index clone +1 (as the number of evolved clones did not include the original index clone).

To abrogate the arbitrary 5%-limit designating the index clones, two indicators of clonal complexity were used for the statistical analysis:

(1) The number of foster clones.
(2) The maximum across all foster clones of the number of evolved clones from one foster clone (referred to as the maximum number of evolved clones hereafter). This number included the foster clone itself.

### Description of the Population and Disease

The following covariates were considered in the description of the population and for the assessment of prognostic categorization: sex, age at diagnosis (1 to <5 vs. 5 to <10 vs. ≥ 10 years), white blood cell (WBC) count at diagnosis (<10 × 10^9^/L vs. 10 × 10^9^ to <50 × 10^9^/L vs. ≥50 × 10^9^/L), initial CNS involvement (CNS-1 vs. others) (30, 31), National Cancer Institute (NCI) risk group (33), EORTC risk group after prephase, and ALL genetic type. EORTC risk group was defined as in the 58951 trial analysis [initial very low risk (VLR) vs. average risk 1 (AR1) vs. AR2 vs. very high risk (VHR)] (30, 31). ALL genetic type was classified as: *t*(12;21)/*ETV6:RUNX1* abnormality determined by FISH vs. hyperdiploidy vs. others. Hyperdiploidy (yes vs. no) was defined as in previous EORTC studies (>50 chromosomes, DNA index, and FISH used in case of no reliable cytogenetic data available) (34).

### Statistical Analysis

We studied the association between clonal complexity as indicated by the number of foster clones and the maximum number of evolved clones and other patients’ characteristics. For the continuous covariates (age and WBC count) and the ordinal covariate EORTC risk group (VLR vs. AR1 vs. AR2 vs. VHR), the Spearman test was used. For the covariates with two categories (sex, NCI risk group, and initial CNS involvement defined as CNS-1 vs. other) and the nominal covariate ALL genetic type [*t*(12;21)/*ETV6:RUNX1* vs. hyperdiploidy vs. other], the Kruskal–Wallis test was used. All tests were performed at a two-sided significance level of 0.05. The analysis was performed using SAS version 9.4.

### Ethical Approval

Agreement for clinical and biological research according to local and international guidelines had been issued at the time of inclusion in the EORTC-58951 clinical trial. DNA samples from leukemic cells were issued from archived material issued for diagnostic and follow-up purposes.

## Results

### Description of the Population

The description of the population is summarized in Table 1. Among the patients from the EORTC-58951 study, 105 who entered the trial between 2004 and 2008 meeting the inclusion criteria of BCP-ALL with the target for the *IgH* study were selected (Supplementary Figure 1). There were 75 patients (71.4%) with NCI standard-risk leukemia and 30 (28.6%) with NCI high-risk leukemia. According to the EORTC risk group, 23 patients (21.9%) were in the VLR group, 63 (60.0%) in the AR1 group, 9 (8.6%) in the AR2 group, and 10 (9.5%) in the VHR group. Leukemia involved *t*(12;21)/*ETV6:RUNX1* abnormality in 27 cases (25.7%) and hyperdiploidy in 39 cases (38.6%).

**TABLE 1:** Description of the population.

	Patients (*N* = 105)
	*N* (%) or median
**Sex**	
Male	60 (57.1)
Female	45 (42.9)
**Age, years**	
Median	4.1
1 to <5	63 (60.0)
5 to <10	25 (23.8)
≥10	17 (16.2)
**WBC, 10^9^/l**	
Median	9.5
<10	55 (52.4)
10 to <50	35 (33.3)
≥50	15 (14.3)
**NCI risk group**	
Standard risk	75 (71.4)
High risk	30 (28.6)
**EORTC risk group after prephase**	
VLR	23 (21.9)
AR1	63 (60.0)
AR2	9 (8.6)
VHR	10 (9.5)
**Initial CNS involvement**	
CNS-1	92 (87.6)
CNS-2/TLP+	11 (10.5)
Missing	2 (1.9)
***t*(12;21)/*ETV6:RUNX1***	
No	75 (71.4)
Yes	27 (25.7)
Missing	3 (2.9)
**Hyperdiploidy**	
No	62 (59.0)
Yes	39 (37.1)
Missing	4 (3.8)
**Hypodiploidy**	
No	94 (89.5)
Yes	2 (1.9)
Missing	9 (8.6)

*WBC, white blood cell; NCI, National Cancer Institute; EORTC, European Organization for Research and Treatment of Cancer; VLR, very low risk; AR-1/2, average risk 1/2; VHR, very high risk; CNS, central nervous system.*

### Descriptive Statistics of the Number of Clones

**FIGURE 2:**
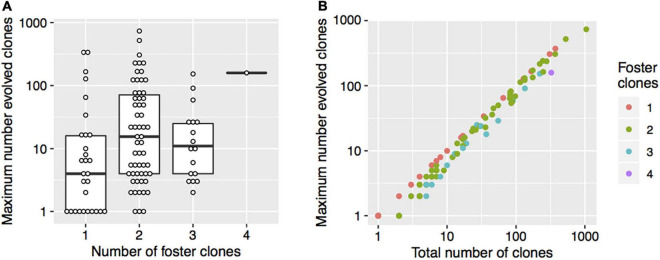
The number of foster clones, the maximum number of evolved clones, and the total number of clones. **(A)** The thick horizontal lines represent the medians and the boxes indicate the first and the third quartiles of the maximum number of evolved clones. Each point shows data for one patient. **(B)** The colors indicate the number of foster clones. Each point shows data for one patient.

The number of index clones was 1 for 24.8%, 2 for 45.7%, 3 for 23.8%, and greater than 3 for 5.7% of the patients (Table 2 and Supplementary Table 1). The number of foster clones was 1 for 27.6%, 2 for 55.2%, 3 for 16.2%, and 4 for 1.0% of the patients. The median of the total number of evolved clones per patient was 13 (range: 1–1,046). The median number of the maximum number of evolved clones was 9 (range: 1–734). The maximum number of evolved clones was weakly associated with the number of foster clones (Spearman correlation: rho = 0.20, *p* = 0.04) (Figure 2A). On the log scale, there was a strong linear association between the total number of clones and the maximum number of evolved clones, indicating that among patients with many evolved clones, the clones typically evolved from the same foster clone (Figure 2B).

**TABLE 2:** Distribution of the number of index clones and foster clones.

	Number of foster clones				
	1 (*N* = 29)	2 (*N* = 58)	3 (*N* = 17)	4 (*N* = 1)	Total (*N* = 105)
			
	*N* (%)	*N* (%)	*N* (%)	*N* (%)	*N* (%)
**Number of index clones**					
1	26 (89.7)	0 (0.0)	0 (0.0)	0 (0.0)	26 (24.8)
2	2 (6.9)	46 (79.3)	0 (0.0)	0 (0.0)	48 (45.7)
3	1 (3.4)	9 (15.5)	15 (88.2)	0 (0.0)	25 (23.8)
4	0 (0.0)	2 (3.4)	2 (11.8)	0 (0.0)	4 (3.8)
5	0 (0.0)	0 (0.0)	0 (0.0)	1 (100.0)	1 (1.0)
8	0 (0.0)	1 (1.7)	0 (0.0)	0 (0.0)	1 (1.0)

### Associations Between Clonal Complexity and Baseline Characteristics of the Patients

We found no significant association between the number of foster clones and the covariates sex, age, WBC count at diagnosis, initial CNS involvement, or NCI risk group (Table 3). Patients in lower EORTC risk groups had more foster clones than patients in higher-risk groups (*p* = 0.007). The genetic type was associated with the number of foster clones as well (*p* = 0.032), with patients with *t*(12;21)/*ETV6:RUNX1* having a smaller number of foster clones. Among patients with *t*(12;21)/*ETV6:RUNX1*, 7.4% of the patients had three or four clones, as compared with 25.6% among patients with hyperdiploidy and 17.1% of patients with other genetic types.

**TABLE 3:** Associations between the number of foster clones and patients’ characteristics.

	Number of foster clones				
	1 (*N* = 29)	2 (*N* = 58)	3 (*N* = 17)	4 (*N* = 1)	*p*-Value
	*N* (row %)	*N* (row %)	*N* (row %)	*N* (row %)	
Sex					0.076
Male	14 (23.3)	32 (53.3)	14 (23.3)	0 (0.0)	
Female	15 (33.3)	26 (57.8)	3 (6.7)	1 (2.2)	
Age, years					0.59
1 to <5	16 (25.4)	37 (58.7)	10 (15.9)	0 (0.0)	
5 to <10	8 (32.0)	13 (52.0)	4 (16.0)	0 (0.0)	
≥10	5 (29.4)	8 (47.1)	3 (17.6)	1 (5.9)	
WBC, 10^9^/l					0.45
<10	12 (21.8)	32 (58.2)	10 (18.2)	1 (1.8)	
10 to <50	12 (34.3)	19 (54.3)	4 (11.4)	0 (0.0)	
≥50	5 (33.3)	7 (46.7)	3 (20.0)	0 (0.0)	
Initial CNS involvement					0.75
Number of observations	28	57	17	1	
CNS-1	25 (27.2)	50 (54.3)	17 (18.5)	0 (0.0)	
CNS-2/TLP+	3 (27.3)	7 (63.6)	0 (0.0)	1 (9.1)	
NCI risk group					0.72
Standard risk	20 (26.7)	44 (58.7)	11 (14.7)	0 (0.0)	
High risk	9 (30.0)	14 (46.7)	6 (20.0)	1 (3.3)	
EORTC risk group after prephase					0.007
VLR	2 (8.7)	14 (60.9)	7 (30.4)	0 (0.0)	
AR1	20 (31.7)	33 (52.4)	9 (14.3)	1 (1.6)	
AR2	2 (22.2)	7 (77.8)	0 (0.0)	0 (0.0)	
VHR	5 (50.0)	4 (40.0)	1 (10.0)	0 (0.0)	
Genetic type					0.032
Number of observations	28	55	17	1	
*t*(12;21)/ *ETV6:RUNX1*	11 (40.7)	14 (51.9)	2 (7.4)	0 (0.0)	
Hyperdiploidy	6 (15.4)	23 (59.0)	10 (25.6)	0 (0.0)	
Other	11 (31.4)	18 (51.4)	5 (14.3)	1 (2.9)	

*WBC, white blood cell; CNS, central nervous system; NCI, National Cancer Institute; EORTC, European Organization for Research and Treatment of Cancer; VLR, very low risk; AR-1, –2, average risk-1, –2; VHR, very high risk.*

**FIGURE 3:**
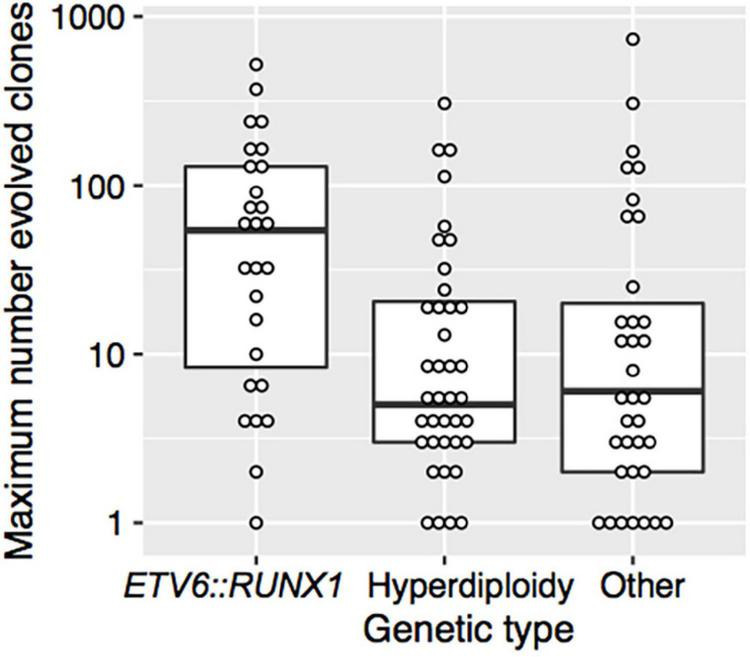
The maximum number of evolved clones vs. genetic type. The thick horizontal lines represent the medians and the boxes indicate the first and the third quartiles of the maximum number of evolved clones. Each point shows data for one patient.

There was no significant association between the covariates sex, age, WBC count at diagnosis, NCI, EORTC risk group, or initial CNS involvement and the maximum number of evolved clones (Table 4). However, the maximum number of evolved clones was strongly associated with the genetic type (*p* = 0.002) (Table 4 and Figure 3). Patients with *t*(12;21)/*ETV6:RUNX1* had significantly more evolved clones than patients with hyperdiploidy or other genetic abnormalities. The median of the maximum number of evolved clones was 54 for *t*(12;21)/*ETV6:RUNX1*, 5 for hyperdiploidy, and 6 for other genetic types.

**TABLE 4:** Associations between the maximum number of evolved clones and patients’ characteristics.

	*N*	Min	Q1	Median	Q3	Max	*p*-Value
Sex							0.59
Male	60	1	3.5	9.5	55.5	734	
Female	45	1	3.0	8.0	64.0	306	
Age, years							0.072
1 to <5	63	1	4.0	13.0	64.0	734	
5 to <10	25	1	4.0	12.0	36.0	172	
≥10	17	1	2.0	3.0	57.0	159	
WBC, 10^9^/l							0.49
<10	55	1	3.0	7.0	50.0	734	
10 to <50	35	1	3.0	17.0	113.0	520	
≥50	15	1	3.0	12.0	36.0	371	
Initial CNS involvement, *N* = 103							0.78
CNS-1	92	1	3.0	9.5	61.5	734	
CNS-2/TLP+	11	1	2.0	9.0	32.0	216	
NCI risk group							0.065
Standard risk	75	1	4.0	13.0	65.0	734	
High risk	30	1	2.0	4.0	36.0	371	
EORTC risk group							0.48
VLR	23	1	3.0	5.0	20.0	153	
AR1	63	1	4.0	16.0	79.0	734	
AR2	9	1	2.0	9.0	36.0	371	
VHR	10	1	1.0	8.5	32.0	172	
Genetic type, *N* = 101							0.002
*t*(12;21)/*ETV6:RUNX1*	27	1	7.0	54.0	131.0	520	
Hyperdiploidy	39	1	3.0	5.0	21.0	306	
Other	35	1	2.0	6.0	25.0	734	

*Min, minimum; Q1, first quartile; Q3, third quartile; Max, maximum; WBC, white blood cell; CNS, central nervous system; NCI, National Cancer Institute; EORTC, European Organization for Research and Treatment of Cancer; VLR, very low risk; AR-1, –2, average risk-1, –2; VHR, very high risk.*

## Discussion

To analyze the correlation between clonal complexity of leukemia at diagnosis and described prognostic risk factors, we chose in this study the categories of foster clones and evolved clones from the foster clone. The notion of index clone, as described since the first studies on *IgH* HTS, takes indeed separately into account each clone with a frequency higher than 5% (3). By manually analyzing the D-N_2_-J sequences, we could gather index clones belonging together, sharing the same D-N_2_-J stem, thus originating from the same foster clone. Then, manual sorting allowed us to describe precisely the number of evolved clones, regardless of their percentage. The numbers of foster clones and of evolved clones from the foster clones were significantly associated. Leukemia with a higher number of foster clones had also more evolved clones.

A majority of patients had two foster clones. This observation was enabled by the fact that we considered the foster clones rather than the index clones and would be in line with Alves-Pereira et al. (35), who showed in 2014 that both *IgH* alleles are recruited independently and in parallel during V(D)J recombination in pre-B cells. This process of rearrangement is regulated by feedback mechanisms that are set up once a productive *V*_*H*_ to *DJ*_*H*_ joining took place and which are partly lacking in leukemic cells (11). It has also been suggested that the pattern of ongoing rearrangements in an individual patient reflects the *IgH* rearrangement status of the precursor cell at the time of malignant transformation (11). As the idea of a monoclonal origin of ALL is nowadays undermined by many studies that reported that up to 40% of BCP-ALL are (at least) oligoclonal at diagnosis (3, 36), this might explain why 18 patients had more than two foster clones (17%). Furthermore, it is not known what proportion of cells rearrange the alleles synchronously (35), which could also account for leukemic cell lines with only one foster clone.

In patients with *t*(12;21)/*ETV6:RUNX1*, there were significantly less foster clones but the maximum number of evolved clones was higher. The *t*(12;21)/*ETV6:RUNX1* translocation is present in around 25% of childhood BCP-ALL (5) and is related to a better prognosis (37). This would therefore be in line with the fact that patients who had more clonal evolutions also belonged more often to the EORTC VLR or AR1 risk.

That patients with *t*(12;21)/*ETV6:RUNX1* had significantly less foster clones, and a higher maximum number of evolved clones could confirm results obtained in 2004 by Hübner et al. on *t*(12;21)/*ETV6:RUNX1* BCP-ALL, who found that *t*(12;21)/*ETV6:RUNX1* BCP-ALL had a higher number of Ig/TCR rearrangements but with lower IgH oligoclonality (38).

Biologically, *t*(12;21)/*ETV6:RUNX1* would appear early in leukemic blasts (39) and lead to an arrest in B-cell differentiation but would not be sufficient to induce leukemia (29). The critical secondary events leading to leukemic transformation in *t*(12;21)/*ETV6:RUNX1* BCP-ALL would frequently be linked to genomic rearrangements mediated by aberrant RAG recombinase activity (27), which is increased in *t*(12;21)/*ETV6:RUNX1* BCP-ALL (40, 41). The fact that RAG activity plays an important role in V(D)J rearrangement (26, 42) might furthermore explain why we found significantly more clonal evolution in the patients who had *t*(12,21)/*ETV6:RUNX1* positive BCP-ALL.

The RAG activity is however not only increased in *t*(12;21)/*ETV6:RUNX1* BCP-ALL and is found, for example, in BCR-ABL1 ALL (28), which were considered of bad prognosis before the availability of tyrosine kinase inhibitors (43). ALL with KMT2A translocation in infants are also known to be oligoclonal and are of worse prognosis (36, 44). Moreover, if RAG activity would be responsible for secondary translocations in *t*(12;21)/*ETV6:RUNX1* BCP-ALL, they seem neither to be the cause of the latter translocation nor explain early translocations in fetal life (45). Besides, the relation between the number of clones and the molecular characteristics of the leukemic cells has not been established so far and relies on much more complex and multifactorial mechanisms than RAG activity, which intervenes at the cleavage phase. In 2014, Gawad et al. (46) individually sequenced 1,479 single tumor cells from six patients with BCP-ALL. In addition to the clonal structure of the disease, they showed how deletions, *IgH* sequences, and specific mutations segregated between clones. They also confirmed (19, 47) that ongoing V(D)J recombination of variable magnitude between different clones in the same patient could occur in the most evolved clones. Generalization of their results might however be difficult, as five out of the six patients in the study harbored *t*(12;21)/*ETV6:RUNX1*.

In their first HTS study (3), Gawad et al. showed the multiplicity of the potential evolution of leukemic cells. We found between 1 and 1,046 evolved clones per patient in our study. This number differed greatly in different studies, between 1 and 4,025 in the study by Gawad et al. (3), between 1 and 6,934 in the study by Faham et al. (17), or between 9 and 59 in the study by Bashford-Rogers et al. (9). Nevertheless, most studies do not refer to the total number of evolved clones, and the phylogeny of the leukemic cells is not always taken into account in HTS studies. The evolution of the IgH repertoire defining the evolved clones as seen by HTS needs however to be studied deeper, as it seems to progress separately from the mutational evolution of subclones (6, 48), or even from the changing immunophenotype of subclones (46) alongside the disease and at relapse (49).

With the generalization of HTS as a new way to efficiently monitor MRD (18), there is all the more a need for the consensual definition for clones and subclones, as MRD is another independent prognostic marker of BCP-ALL, if not the most important (12, 13, 15). A European network, the EuroClonality-NGS Consortium was created to tackle these questions (23) as software are being designed and tested (20, 22, 50, 51) for monitoring MRD by HTS. This study gives insight into the problems that have to be considered whenever leukemic clones are defined by their clonal *IgH* sequence. The question remains whether evolved clones are part of the leukemic clone and thus have to be monitored in the MRD testing or just an epiphenomenon reflecting the maturation phase of the leukemic clone. Moreover, we based our study on index clones (and consecutively, on foster clones) defined as clones above the threshold of five percent of the individual clonotypes (3, 32). With evolving HTS technics and deeper sequencing, this definition could also vary and lead to another comprehension of the clonal landscape of leukemias.

Finally, we found no statistically significant association between the other characteristics and prognostic factors for leukemia in children (sex, age, WBC count at diagnosis, NCI risk group, or initial CNS involvement) and the number of foster clones and evolved clones. One possible explanation for this is the relatively small number of cases investigated. Although this study constitutes one of the biggest cohorts on *IgH* HTS at diagnosis, the sample size would not have allowed enough statistical power to analyze the association between clonal complexity and EFS or other described prognostic factors of pediatric leukemia, which is indeed a very heterogeneous disease with many prognostic factors present only in small subsets of patients, as, for example, genetic abnormalities (52, 53). Furthermore, only patients with *IgH* recombinations were included in this study and we did not investigate the association between the absence of *IgH* recombination (which stipulated an exclusion from our cohort) and belonging to a particular group of risk. Ding et al., among others, suggested that it might be interesting to also look at recombinations of the TCR, even in patients with BCP-ALL, as around 10% of their cohort of patients with BCP-ALL expressed a dominant TCR rearrangement (24 cases out of 219 patients with ALL) (49), while the so-called illegitimate rearrangements – TR rearrangements in BCP-ALL – have been identified in up to 80–90% of patients with BCP-ALL (54, 55). Likewise, some 40% of BCP-ALL also carry an *IGK* rearrangement (54). We focused our study on diagnostic blasts as sequencing of relapse samples was not available to us. Studies targeting the evaluation of HTS for MRD monitoring will hopefully allow gaining access to such data (20).

Furthermore, we limited our study to IgH sequencing and did not look at BCR expression. It has been shown that many ALL carry non-productive *BCR/TCR* (56) in both alleles or the only expressed dominant allele, which was suggested to support the hypothesis that BCR might act as a tumor suppressor in most cases of B-precursor ALL (57). This might therefore also be looked at when considering prognostic factors and *IgH* rearrangements.

One interesting point in our study remains that we considered the number of clones and evolved clones as a potential individual and isolated prognostic marker of the disease, a question not referred to in studies on clonality in BCP-ALL. There is to date less knowledge of the link between genetic alterations in BCP-ALL and recombinations of the *IgH* or *TCR*, although some authors suggested a role of some genetic aberrations or age at diagnosis (38, 55, 58, 59). New methods to dig into clonality as single-cell DNA amplicon sequencing (60) could help understand those mechanisms and be combined with sequencing of the *IgH* or *TCR*.

Our study does not allow the proclamation of the number of foster clones or of evolved clones from the foster clone as new and prognostic factors for childhood BCP-ALL. Further studies on a bigger scale would be needed to support this hypothesis and might end up in subclonal analyses being part of compound prognostic scores. The generalization of HTS methods for the measurement of MRD might bring opportunities to gain access to such HTS data of diagnostic and follow-up ALL samples.

## Data Availability Statement

The datasets presented in this study can be found in online repositories. The names of the repository/repositories and accession number(s) can be found below: https://github.com/GabrielLevyUCLicr/LevyBakkusEtAl_frontiers.

## Ethics Statement

The studies involving human participants were reviewed and approved by the clinical trial registered: https://clinicaltrials.gov/ct2/show/NCT00003728. Written informed consent to participate in this study was provided by the participants’ legal guardian/next of kin.

## Author Contributions

GL, MK, BB, and MB designed the study, interpreted the data, and wrote the manuscript. JV and MB performed the experiments. MK performed the statistical analysis. AU, AF, BD, M-FD, and CC provided data on patients. All authors contributed to the article and approved the submitted version.

## Conflict of Interest

The authors declare that the research was conducted in the absence of any commercial or financial relationships that could be construed as a potential conflict of interest.

## Publisher’s Note

All claims expressed in this article are solely those of the authors and do not necessarily represent those of their affiliated organizations, or those of the publisher, the editors and the reviewers. Any product that may be evaluated in this article, or claim that may be made by its manufacturer, is not guaranteed or endorsed by the publisher.
