# Supplementary figures and images for: Immunoglobulin Heavy Chain High-Throughput Sequencing in Pediatric B-Precursor Acute Lymphoblastic Leukemia: Is the Clonality of the Disease at Diagnosis Related to Its Prognosis?
Source: Front Pediatr. 2022 May 30;10:874771. doi: 10.3389/fped.2022.874771 (PMC9197340; doi:10.3389/fped.2022.874771)

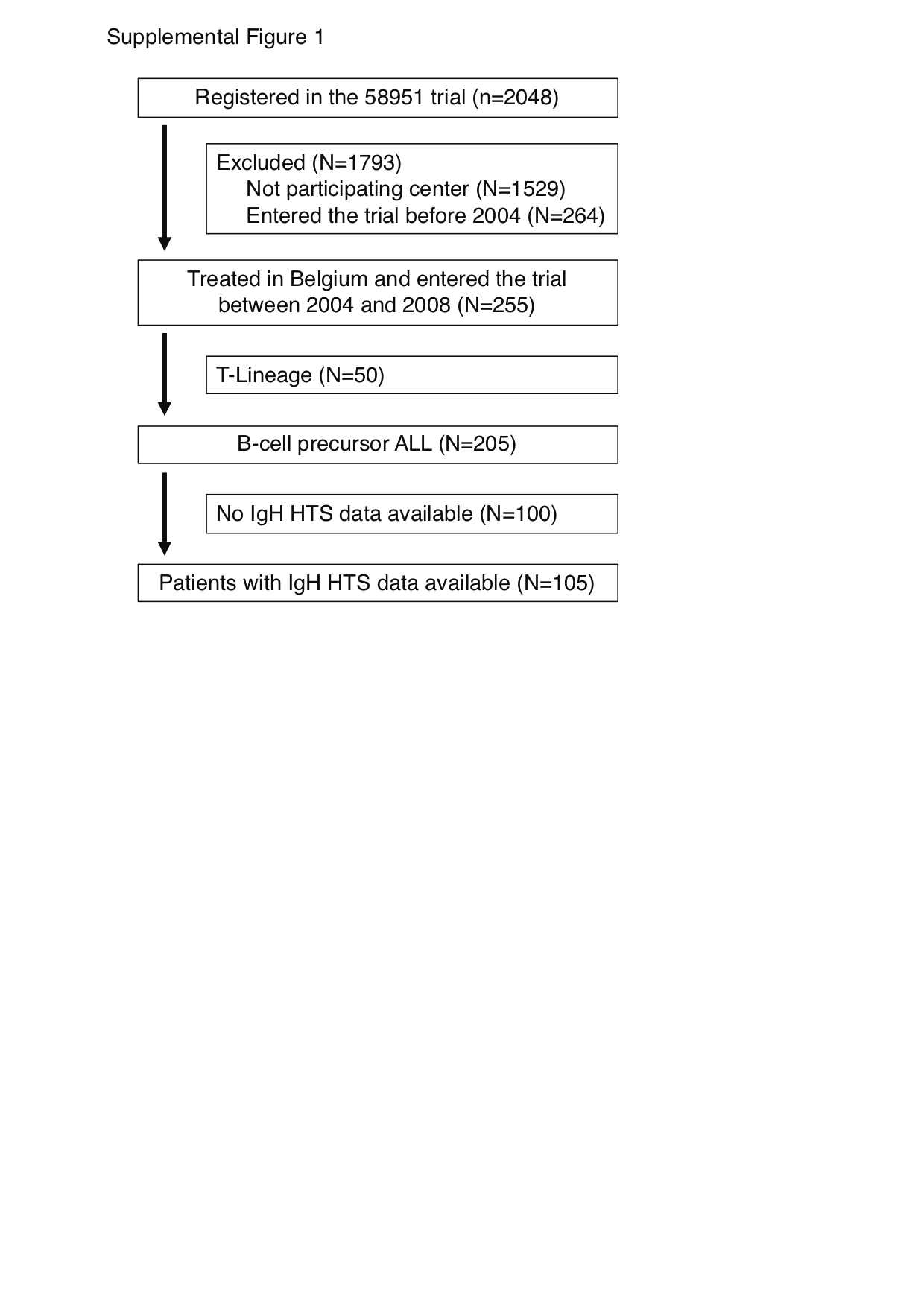

Supplement: Supplementary file 1 [file Image_1.JPEG]
