# Supplementary material for: Immunoglobulin Heavy Chain High-Throughput Sequencing in Pediatric B-Precursor Acute Lymphoblastic Leukemia: Is the Clonality of the Disease at Diagnosis Related to Its Prognosis?
Source: Front Pediatr. 2022 May 30;10:874771. doi: 10.3389/fped.2022.874771 (PMC9197340; doi:10.3389/fped.2022.874771)
Supplement: Supplementary file 2 [file Table_1.DOCX]

Supplemental Table 1: Repartition of the index clones, foster clones, evolved clones of the foster clones and total number of clones in the population with the IgH V(D)J sequences of the index clones

| N° patient | Number of index clones per patient | Index clone indicator | IgH VH seq analysis | Number of foster clones per patient | Foster clone indicator | Evolved clones of the foster clones | Number of clones of the foster clone | Total number of clones per patient | Total number of clones per foster clone |
| --- | --- | --- | --- | --- | --- | --- | --- | --- | --- |
| 1 | 4 | 1 | V3-64*01 0 / +15 / -4 D3-22*01 -12 / +5 / -4 J3*02 | 2 | 3 | 0 | 1 | 84 | 82 |
|  |  | 2 | V1-3*01 -2 / +5 / 0 D3-10*01 -7 / +16 / 0 J6*02 |  | 2 | 1 | 2 |  | 2 |
|  |  | 3 | V4-59*07 -1 / +10 / -4 D3-22*01 -12 / +5 / -4 J3*02 |  | 3 | 79 | 80 |  |  |
|  |  | 4 | V3-64*01 -9 / +24 / -4 D3-22*01 -12 / +5 / -4 J3*02 |  | 3 | 0 | 1 |  |  |
| 2 | 3 | 1 | V3-15*02 -1 / +6 / -7 D2-2*03 -12 / +16 / -15 J4*02 | 2 | 1 | 1 | 2 | 174 | 2 |
|  |  | 2 | V3-33*01 0 / +8 / -5 D2-15*01 0 / +11 / -13 J4*02 |  | 3 | 0 | 1 |  | 172 |
|  |  | 3 | V1-3*01 -3 / +5 / -5 D2-15*01 0 / +11 / -13 J4*02 |  | 3 | 170 | 171 |  |  |
| 3 | 1 | 1 | V4-34*02 -1 / +4 / 0 D3-22*01 -6 / +8 / -16 J4*02 | 1 | 1 | 2 | 3 | 3 | 3 |
| 4 | 2 | 1 | V3-74*02 0 / +13 / 0 D2-8*01 -3 / +16 / -21 J5*02 | 2 | 1 | 21 | 22 | 24 | 22 |
|  |  | 2 | V3-13*01 -1 / +6 / -2 D6-13*01 0 / 0 / -1 J2*01 |  | 2 | 1 | 2 |  | 2 |
| 5 | 3 | 1 | V3-G*01 -16 / +2 / -4 J6*02 | 3 | 1 | 0 | 1 | 5 | 1 |
|  |  | 2 | V3-7*01 -2 / +17 / -2 D3-22*01 -8 / +9 / -1 J5*02 |  | 2 | 2 | 3 |  | 3 |
|  |  | 3 | V6-1*02 -14 / +7 / 0 D2-2*02 -5 / +6 / -6 J4*02 |  | 3 | 0 | 1 |  | 1 |
| 6 | 2 | 1 | V3-64*01 -15 / +12 / -2 D6-6*01 0 / 0 / -4 J1*01 | 2 | 1 | 2 | 3 | 244 | 3 |
|  |  | 2 | V3-30*18 0 / +4 / -1 D6-13*01 -2 / 0 / -5 J4*02 |  | 2 | 240 | 241 |  | 241 |
| 7 | 3 | 1 | V3-7*01 -2 / +11 / -4 D2-2*03 -4 / +20 / -5 J6*02 | 2 | 2 | 0 | 1 | 88 | 31 |
|  |  | 2 | V3-23*04 -7 / +29 / -5 D2-2*03 -4 / +20 / -5 J6*02 |  | 2 | 29 | 30 |  | 57 |
|  |  | 3 | V3-7*01 0 / +7 / -10 D3-9*01 -7 / +11 / -2 J6*02 |  | 3 | 56 | 57 |  |  |
| 8 | 2 | 1 | V3-33*01 0 / +11 / 0 D6-6*01 -1 / +6 / 0 J3*02 | 2 | 1 | 83 | 84 | 247 | 84 |
|  |  | 2 | V1-14*01 0 / +5 / -16 D2-2*03 -23 / 0 / -10 J6*03 |  | 2 | 162 | 163 |  | 163 |
| 9 | 1 | 1 | V3-11*01 -1 / +6 / -2 D3-22*01 -5 / 0 / -5 J4*02 | 1 | 1 | 7 | 8 | 8 | 8 |
| 10 | 2 | 1 | V3-13*01 -3 / +13 / -17 J6*02 | 2 | 1 | 3 | 4 | 5 | 4 |
|  |  | 2 | V3-13*01 -1 / +23 / -4 J4*02 |  | 2 | 0 | 1 |  | 1 |
| 11 | 1 | 1 | V3-71*01 -1 / +31 / -4 D1-26*01 -3 / +1 / -3 J5*02 | 1 | 1 | 2 | 3 | 3 | 3 |
| 12 | 3 | 1 | V4-34*02 -1 / +4 / -22 J5*02 | 2 | 1 | 2 | 3 | 23 | 3 |
|  |  | 2 | V1-8*01 -1 / +5 / -8 D6-13*01 -1 / 0 / -6 J4*02 |  | 3 | 0 | 1 |  | 20 |
|  |  | 3 | V4-59*08 -14 / +17 / 0 D6-13*01 -1 / 0 / -6 J4*02 |  | 3 | 18 | 19 |  |  |
| 13 | 2 | 1 | V1-2*04 0 / +9 / -9 D3-10*01 -6 / 0 / -7 J4*02 | 2 | 1 | 1 | 2 | 18 | 2 |
|  |  | 2 | V6-1*02 -5 / +17 / -7 D3-9*01 -12 / +1 / -16 J5*02 |  | 2 | 15 | 16 |  | 16 |
| 14 | 2 | 1 | V3-13*01 0 / +5 / -7 D3-3*01 -3 / 0 / -5 J4*02 | 2 | 1 | 16 | 17 | 81 | 17 |
|  |  | 2 | V3-53*02 -1 / +6 / -8 J4*02 |  | 2 | 63 | 64 |  | 64 |
| 15 | 2 | 1 | V6-1*02 -2 / +13 / 0 D2-2*02 -5 / +21 / -13 D3-22*01 0 / +6 / -5 J6*02 | 2 | 1 | 5 | 6 | 7 | 6 |
|  |  | 2 | V1-17*02 -2 / +13 / -11 D2-2*02 0 / 0 / -4 J4*02 |  | 2 | 0 | 1 |  | 1 |
| 16 | 1 | 1 | V1-2*04 0 / +16 / -8 D3-9*01 -4 / +3 / -12 J4*02 | 1 | 1 | 165 | 166 | 166 | 166 |
| 17 | 3 | 1 | V1-3*01 0 / +14 / -10 D2-8*01 -1 / 0 / -14 D3/OR15-3B*01 -8 / +6 / -13 J4*02 | 1 | 1 | 13 | 14 | 16 | 16 |
|  |  | 2 | V1-2*02 0 / +4 / -5 D2-8*01 -1 / 0 / -14 D3/OR15-3B*01 -8 / +6 / -13 J4*02 |  | 1 | 0 | 1 |  |  |
|  |  | 3 | V4-34*02 -13 / +4 / -13 D2-8*01 -1 / +2 / -3 J5*02 |  | 1 | 0 | 1 |  |  |
| 18 | 1 | 1 | V4-28*02 -8 / +3 / -8 D3-22*01 -1 / 0 / -2 J4*02 | 1 | 1 | 9 | 10 | 10 | 10 |
| 19 | 4 | 1 | V3-22*02 -1 / +5 / -6 D3-3*01 -7 / +9 / -5 D1-7*01 0 / +2 / 0 J4*02 | 3 | 1 | 1 | 2 | 10 | 3 |
|  |  | 2 | V3-30*01 0 / +5 / -14 D3/OR15-3B*01 -8 / 0 / -3 J5*02 |  | 2 | 5 | 6 |  | 6 |
|  |  | 3 | V3-13*01 0 / +12 / -5 D3-9*01 -8 / +5 / -14 D2/OR15-2B*01 -7 / 0 / 0 J4*02 |  | 3 | 0 | 1 |  | 1 |
|  |  | 4 | V3-23*04 0 / +134 / -6 D3-3*01 -7 / +23 / 0 J4*02 |  | 1 | 0 | 1 |  |  |
| 20 | 3 | 1 | V3-23*04 -5 / +19 / 0 J4*02 | 3 | 1 | 3 | 4 | 6 | 4 |
|  |  | 2 | V4-34*02 -5 / +2 / -4 D6-6*01 -1 / +1 / -26 J6*04 |  | 2 | 0 | 1 |  | 1 |
|  |  | 3 | V1-2*04 0 / +7 / -3 D7-27*01 0 / 0 / -2 J2*01 |  | 3 | 0 | 1 |  | 1 |
| 21 | 2 | 1 | V3-64*02 -3 / +6 / -5 D3-22*01 0 / 0 / -7 J6*03 | 2 | 1 | 0 | 1 | 4 | 1 |
|  |  | 2 | V4-31*03 -3 / +15 / 0 D3-10*01 -4 / 0 / 0 J6*03 |  | 2 | 2 | 3 |  | 3 |
| 22 | 2 | 1 | V3-30*18 -2 / +6 / -5 J5*02 | 2 | 1 | 1 | 2 | 6 | 2 |
|  |  | 2 | V4-31*03 0 / +14 / -4 J3*02 |  | 2 | 3 | 4 |  | 4 |
| 23 | 3 | 1 | V3-33*01 -1 / +4 / 0 D3-3*01 -8 / 0 / -4 J4*02 | 2 | 1 | 30 | 31 | 85 | 31 |
|  |  | 2 | V1-2*02 -3 / +28 / -4 J4*02 |  | 3 | 1 | 2 |  | 54 |
|  |  | 3 | V2-5*01 0 / +34 / -4 J4*02 |  | 3 | 51 | 52 |  |  |
| 24 | 2 | 1 | V3-33*01 -3 / +5 / -3 D6-6*01 0 / 0 / 0 J2*01 | 2 | 1 | 0 | 1 | 2 | 1 |
|  |  | 2 | V5-51*01 -1 / +4 / -2 D4-23*01 -5 / +2 / -4 J4*02 |  | 2 | 0 | 1 |  | 1 |
| 25 | 2 | 1 | V3-23*04 -2 / +4 / 0 D2-2*02 -1 / +35 / -5 J6*03 | 2 | 1 | 0 | 1 | 3 | 1 |
|  |  | 2 | V2-10*01 -3 / +5 / -2 D5-5*01 0 / +19 / -6 J4*02 |  | 2 | 1 | 2 |  | 2 |
| 26 | 2 | 1 | V4-30-4*01 -3 / +8 / -5 D1-7*01 0 / 0 / -3 J6*02 | 2 | 1 | 8 | 9 | 45 | 9 |
|  |  | 2 | V4-55*01 -1 / +41 / -8 D2-2*02 -1 / 0 / -1 J1*01 |  | 2 | 35 | 36 |  | 36 |
| 27 | 2 | 1 | V3-33*01 -5 / +19 / -4 J4*02 | 2 | 1 | 7 | 8 | 12 | 8 |
|  |  | 2 | V3-11*01 0 / +15 / -7 D3-3*01 -1 / 0 / -5 J1*01 |  | 2 | 3 | 4 |  | 4 |
| 28 | 2 | 1 | V3-33*01 -5 / +7 / -5 D2-8*01 -2 / 0 / -4 J1*01 | 2 | 1 | 1 | 2 | 4 | 2 |
|  |  | 2 | V3-9*01 0 / +12 / -10 D3-10*01 -7 / 0 / -8 J6*02 |  | 2 | 1 | 2 |  | 2 |
| 29 | 1 | 1 | V1-69*13 -5 / +22 / -11 D3-10*02 -2 / +1 / -5 J4*02 | 1 | 1 | 370 | 371 | 371 | 371 |
| 30 | 2 | 1 | V4-34*02 -8 / +9 / -4 D2-21*02 -4 / +8 / -4 J4*02 | 2 | 1 | 1 | 2 | 4 | 2 |
|  |  | 2 | V6-1*02 0 / +8 / -13 D3-22*01 0 / 0 / -7 J6*02 |  | 2 | 1 | 2 |  | 2 |
| 31 | 2 | 1 | V3-7*01 -1 / +5 / -7 D6-13*01 0 / 0 / 0 J5*02 | 2 | 1 | 38 | 39 | 276 | 39 |
|  |  | 2 | V4-59*07 0 / +8 / -19 D3-22*01 0 / 0 / -3 J5*02 |  | 2 | 236 | 237 |  | 237 |
| 32 | 2 | 1 | V3-30-3*01 -1 / +24 / -8 J4*02 | 2 | 1 | 22 | 23 | 36 | 23 |
|  |  | 2 | V1-2*04 0 / 0 / -1 D4-17*01 -1 / 0 / 0 J4*02 |  | 2 | 12 | 13 |  | 13 |
| 33 | 2 | 1 | V3-7*01 -1 / +13 / -9 D6-13*01 0 / +4 / -1 J4*02 | 2 | 1 | 23 | 24 | 30 | 24 |
|  |  | 2 | V3-13*01 0 / +22 / -10 D3-3*02 -4 / 0 / 0 J6*03 |  | 2 | 5 | 6 |  | 6 |
| 34 | 2 | 1 | V3-15*02 -5 / +25 / 0 J5*02 | 2 | 1 | 1 | 2 | 115 | 2 |
|  |  | 2 | V3-7*01 -3 / +45 / -1 D4-17*01 0 / +14 / -6 J6*02 |  | 2 | 112 | 113 |  | 113 |
| 35 | 3 | 1 | V1-3*01 -1 / +9 / 0 D4-17*01 0 / 0 / 0 J4*02 | 2 | 1 | 2 | 3 | 6 | 4 |
|  |  | 2 | V1-58*02 0 / +35 / -5 D3-3*01 -1 / 0 / 0 J6*04 |  | 2 | 1 | 2 |  | 2 |
|  |  | 3 | V4-4*02 -8 / +13 / -3 D4-17*01 0 / 0 / 0 J4*02 |  | 1 | 0 | 1 |  |  |
| 36 | 2 | 1 | V1-46*01 0 / +8 / -3 D3-3*01 -2 / 0 / 0 J6*03 | 2 | 1 | 3 | 4 | 7 | 4 |
|  |  | 2 | V3-11*01 -13 / +9 / -12 D3-3*02 -2 / 0 / 0 J6*03 |  | 2 | 2 | 3 |  | 3 |
| 37 | 2 | 1 | V3-9*01 0 / +19 / -4 J3*02 | 2 | 1 | 0 | 1 | 6 | 1 |
|  |  | 2 | V3-9*01 -19 / +7 / 0 D2-15*01 -1 / 0 / -3 J1*01 |  | 2 | 4 | 5 |  | 5 |
| 38 | 3 | 1 | V3-73*02 -1 / +3 / -4 D2-8*01 -4 / +4 / -6 J6*03 | 2 | 1 | 1 | 2 | 47 | 2 |
|  |  | 2 | V6-1*02 -1 / +3 / -3 D1-1*01 -2 / +4 / -3 J3*02 |  | 2 | 43 | 44 |  | 45 |
|  |  | 3 | V4-34*02 0 / +15 / -3 J3*02 |  | 2 | 0 | 1 |  |  |
| 39 | 2 | 1 | V4-34*02 -5 / +12 / -16 D2-2*03 -22 / 0 / -7 J6*02 | 2 | 1 | 3 | 4 | 9 | 4 |
|  |  | 2 | V4-34*02 -3 / +6 / -8 D2-21*02 -4 / +2 / -10 J6*02 |  | 2 | 4 | 5 |  | 5 |
| 40 | 1 | 1 | V3-74*02 -1 / +12 / -6 D2-2*03 -3 / 0 / -6 J4*02 | 1 | 1 | 6 | 7 | 7 | 7 |
| 41 | 1 | 1 | V6-1*02 -2 / +2 / -3 D6-6*01 -1 / +4 / -2 J4*02 | 1 | 1 | 15 | 16 | 16 | 16 |
| 42 | 1 | 1 | V3-22*02 -3 / +5 / -4 D2-8*01 -4 / +11 / -5 J6*02 | 1 | 1 | 0 | 1 | 1 | 1 |
| 43 | 3 | 1 | V3-13*01 -1 / +4 / -3 D4-23*01 -1 / +5 / -15 J6*02 | 3 | 1 | 2 | 3 | 6 | 3 |
|  |  | 2 | V7-27*01 -2 / +30 / -10 J4*02 |  | 2 | 0 | 1 |  | 1 |
|  |  | 3 | V6-1*02 0 / +3 / -6 D2-2*03 -6 / +10 / -4 J6*02 |  | 3 | 1 | 2 |  | 2 |
| 44 | 2 | 1 | V3-7*01 0 / +11 / -2 D3-10*01 0 / +15 / -8 J4*02 | 2 | 1 | 4 | 5 | 7 | 5 |
|  |  | 2 | V3-21*02 0 / +23 / -1 D3-3*01 -3 / +27 / 0 J6*02 |  | 2 | 1 | 2 |  | 2 |
| 45 | 1 | 1 | V3-7*01 0 / +12 / 0 D2-8*01 -6 / +28 / -12 J6*02 | 1 | 1 | 0 | 1 | 1 | 1 |
| 46 | 2 | 1 | V3-30-3*01 0 / +10 / -5 D3-9*01 -6 / +2 / -4 J4*02 | 2 | 1 | 39 | 40 | 174 | 40 |
|  |  | 2 | V4-34*02 0 / +12 / -3 D3-22*01 -13 / +12 / -7 J4*02 |  | 2 | 133 | 134 |  | 134 |
| 47 | 1 | 1 | V3-13*01 0 / 0 / -5 D2-21*02 -8 / +2 / -1 J5*02 | 1 | 1 | 3 | 4 | 4 | 4 |
| 48 | 1 | 1 | V3-41*01 0 / +6 / -3 D4-4*01 -2 / 0 / -3 J4*02 | 1 | 1 | 5 | 6 | 6 | 6 |
| 49 | 2 | 1 | V4-39*01 -3 / +23 / 0 J4*02 | 2 | 1 | 8 | 9 | 13 | 9 |
|  |  | 2 | V1-69*13 -3 / +6 / -2 D7-27*01 0 / +6 / -6 J4*02 |  | 2 | 3 | 4 |  | 4 |
| 50 | 4 | 1 | V4-31*03 0 / +4 / 0 D3-10*01 -9 / +6 / -4 J5*02 | 3 | 1 | 12 | 13 | 90 | 14 |
|  |  | 2 | V3-9*01 0 / +6 / -9 D6-19*01 -1 / +4 / -5 J4*02 |  | 2 | 58 | 59 |  | 59 |
|  |  | 3 | V3-13*01 -1 / +5 / 0 D3-10*01 -9 / +6 / -4 J5*02 |  | 1 | 0 | 1 |  | 17 |
|  |  | 4 | V4-4*02 -2 / +7 / -1 D2-15*01 -3 / +5 / -2 J5*02 |  | 4 | 16 | 17 |  |  |
| 51 | 2 | 1 | V3-13*01 -1 / +16 / -8 D2-2*03 -6 / 0 / -1 J5*02 | 2 | 1 | 0 | 1 | 2 | 1 |
|  |  | 2 | V3-13*01 0 / +4 / -4 D6-13*01 -1 / +5 / -1 J6*02 |  | 2 | 0 | 1 |  | 1 |
| 52 | 2 | 1 | V4-59*07 0 / +44 / -5 J4*02 | 2 | 1 | 49 | 50 | 55 | 50 |
|  |  | 2 | V3-64*05 -5 / +20 / -10 D2-21*02 -1 / +3 / -4 J4*02 |  | 2 | 4 | 5 |  | 5 |
| 53 | 8 | 1 | V6-1*02 -1 / +9 / -7 D6-6*01 -1 / +20 / -5 J4*02 | 2 | 1 | 9 | 10 | 133 | 11 |
|  |  | 2 | V4-4*07 0 / +5 / 0 D3-3*01 -8 / +6 / -5 J5*02 |  | 5 | 4 | 5 |  | 122 |
|  |  | 3 | V3-7*01 0 / +5 / -3 D3-3*01 -8 / +6 / -5 J5*02 |  | 5 | 1 | 2 |  |  |
|  |  | 4 | V4-4*07 -1 / +6 / 0 D3-3*01 -8 / +6 / -5 J5*02 |  | 5 | 0 | 1 |  |  |
|  |  | 5 | V6-1*02 0 / +5 / 0 D3-3*01 -8 / +6 / -5 J5*02 |  | 5 | 111 | 112 |  |  |
|  |  | 6 | V4-4*07 -1 / +5 / -2 D3-3*01 -8 / +6 / -5 J5*02 |  | 5 | 0 | 1 |  |  |
|  |  | 7 | V3-7*01 -1 / +3 / -6 D6-6*01 -1 / +20 / -5 J4*02 |  | 1 | 0 | 1 |  |  |
|  |  | 8 | V6-1*02 0 / +7 / -5 D3-3*01 -8 / +6 / -5 J5*02 |  | 5 | 0 | 1 |  |  |
| 54 | 3 | 1 | V3-33*01 -1 / +4 / -7 D6-13*01 -4 / +3 / -1 J1*01 | 3 | 1 | 36 | 37 | 221 | 37 |
|  |  | 2 | V1-3*02 -3 / +4 / -4 D6-13*01 -4 / 0 / -2 J5*02 |  | 2 | 30 | 31 |  | 31 |
|  |  | 3 | V3-7*01 -2 / +4 / -6 D5-5*01 -3 / +1 / -4 D2-8*01 -7 / +7 / -1 J6*02 |  | 3 | 152 | 153 |  | 153 |
| 55 | 2 | 1 | V3-15*02 -16 / +5 / -7 D2-21*02 -2 / +1 / -2 J3*02 | 2 | 1 | 0 | 1 | 2 | 1 |
|  |  | 2 | V3-43*01 -3 / +18 / -5 J4*02 |  | 2 | 0 | 1 |  | 1 |
| 56 | 3 | 1 | V4-4*07 -11 / +10 / -13 D3-10*02 -2 / 0 / -2 J6*02 | 3 | 1 | 2 | 3 | 31 | 3 |
|  |  | 2 | V1-18*01 -1 / 0 / -6 D3-9*01 -4 / 0 / -2 J4*02 |  | 2 | 3 | 4 |  | 4 |
|  |  | 3 | V1-2*02 0 / +9 / -18 D2-2*03 -2 / +10 / -2 J5*02 |  | 3 | 23 | 24 |  | 24 |
| 57 | 2 | 1 | V1-2*04 0 / +11 / -4 J3*02 | 2 | 1 | 4 | 5 | 26 | 5 |
|  |  | 2 | V1-8*01 -1 / +9 / -1 D7-27*01 -1 / +3 / -1 J3*02 |  | 2 | 20 | 21 |  | 21 |
| 58 | 2 | 1 | V3-74*02 0 / +1 / -9 J6*02 | 2 | 1 | 1 | 2 | 6 | 2 |
|  |  | 2 | V3-73*02 -1 / +26 / -4 J4*02 |  | 2 | 3 | 4 |  | 4 |
| 59 | 3 | 1 | V3-21*02 -23 / +17 / -13 J5*02 | 3 | 1 | 0 | 1 | 13 | 1 |
|  |  | 2 | V3-48*02 -3 / +8 / -15 D4-4*01 -8 / 0 / 0 J4*02 |  | 2 | 8 | 9 |  | 9 |
|  |  | 3 | V4-34*02 -7 / +1 / -16 D2-2*02 -2 / +4 / -4 D5-24*01 0 / +4 / -6 J6*02 |  | 3 | 2 | 3 |  | 3 |
| 60 | 2 | 1 | V4-4*07 0 / +3 / -5 D4-4*01 0 / +3 / -6 J6*02 | 2 | 1 | 11 | 12 | 17 | 12 |
|  |  | 2 | V1-3*01 -1 / +6 / 0 D7-27*01 0 / +1 / -10 J4*02 |  | 2 | 4 | 5 |  | 5 |
| 61 | 1 | 1 | V3-66*01 -1 / +22 / -1 J6*03 | 1 | 1 | 0 | 1 | 1 | 1 |
| 62 | 2 | 1 | V3-53*02 -11 / +10 / -10 J4*02 | 2 | 1 | 0 | 1 | 5 | 1 |
|  |  | 2 | V5-51*01 -1 / +12 / -3 D2-8*01 -2 / +40 / -7 J4*02 |  | 2 | 3 | 4 |  | 4 |
| 63 | 2 | 1 | V1-3*01 0 / +2 / -7 D2-21*01 | 2 | 1 | 305 | 306 | 368 | 306 |
|  |  | 2 | V1-46*01 -1 / +5 / -10 D3-10*01 -4 / 0 / -1 J6*02 |  | 2 | 61 | 62 |  | 62 |
| 64 | 3 | 1 | V3-30-3*01 0 / +23 / -4 J4*02 | 2 | 1 | 30 | 31 | 36 | 32 |
|  |  | 2 | V1-2*02 -3 / +11 / -4 J4*02 |  | 1 | 0 | 1 |  | 4 |
|  |  | 3 | V1-2*04 0 / +5 / -10 D2-8*01 -2 / +11 / -5 J6*02 |  | 3 | 3 | 4 |  |  |
| 65 | 2 | 1 | V3-74*02 -10 / +10 / -7 D3-10*01 -5 / +2 / 0 J4*02 | 2 | 1 | 3 | 4 | 524 | 4 |
|  |  | 2 | V3-7*01 0 / +6 / -5 D5-5*01 0 / 0 / -5 J6*02 |  | 2 | 519 | 520 |  | 520 |
| 66 | 1 | 1 | V4-34*02 -3 / +13 / -11 D2-2*03 0 / +10 / -7 J6*02 | 1 | 1 | 0 | 1 | 1 | 1 |
| 67 | 2 | 1 | V1-3*02 -5 / +9 / -18 D2-15*01 -1 / +3 / -3 D1-26*01 0 / +5 / -6 J4*02 | 2 | 1 | 4 | 5 | 14 | 5 |
|  |  | 2 | V4-4*01 -5 / +23 / 0 J6*02 |  | 2 | 8 | 9 |  | 9 |
| 68 | 2 | 1 | V3-30*04 -1 / +29 / -5 J4*02 | 2 | 1 | 14 | 15 | 17 | 15 |
|  |  | 2 | V6-1*02 -3 / +30 / -4 J5*02 |  | 2 | 1 | 2 |  | 2 |
| 69 | 1 | 1 | V4-55*01 -1 / +6 / -3 D3-3*01 -3 / 0 / 0 J6*02 | 1 | 1 | 33 | 34 | 34 | 34 |
| 70 | 1 | 1 | V3-7*01 0 / +1 / -3 D6-19*01 -7 / +11 / -5 J4*02 | 1 | 1 | 3 | 4 | 4 | 4 |
| 71 | 2 | 1 | V1-2*04 0 / +12 / -9 D2-2*02 -2 / +5 / -2 J6*02 | 1 | 1 | 15 | 16 | 17 | 17 |
|  |  | 2 | V1-69*06 -3 / +4 / -14 D2-2*02 -2 / +5 / -2 J6*02 |  | 1 | 0 | 1 |  |  |
| 72 | 3 | 1 | V4-61*02 -2 / +11 / -1 D2-2*03 -6 / 0 / -11 J1*01 | 2 | 1 | 311 | 312 | 1046 | 312 |
|  |  | 2 | V2-5*10 -4 / +13 / -3 J6*02 |  | 3 | 0 | 1 |  | 734 |
|  |  | 3 | V3-7*01 0 / +11 / -3 J6*02 |  | 3 | 732 | 733 |  |  |
| 73 | 2 | 1 | V4-39*01 0 / +20 / -1 D2-8*01 0 / 0 / -4 J5*02 | 2 | 1 | 2 | 3 | 5 | 3 |
|  |  | 2 | V4-55*05 -7 / +17 / -3 J6*02 |  | 2 | 1 | 2 |  | 2 |
| 74 | 1 | 1 | V1-2*02 0 / +10 / -5 D2-2*02 -1 / 0 / -5 J1*01 | 1 | 1 | 305 | 306 | 306 | 306 |
| 75 | 1 | 1 | V3-30*01 -16 / +8 / -10 D3-9*01 -10 / +19 / -10 J4*02 | 1 | 1 | 1 | 2 | 2 | 2 |
| 76 | 2 | 1 | V1-69*09 -2 / +9 / -2 J6*03 | 2 | 1 | 0 | 1 | 4 | 1 |
|  |  | 2 | V2-26*01 -6 / +20 / -2 D6-13*01 0 / 0 / -2 J6*03 |  | 2 | 2 | 3 |  | 3 |
| 77 | 1 | 1 | V3-11*01 0 / +7 / -4 D3-10*01 -9 / +1 / -3 J6*02 | 1 | 1 | 5 | 6 | 6 | 6 |
| 78 | 1 | 1 | V6-1*02 -8 / +11 / -1 D7-27*01 0 / +5 / -18 J5*02 | 1 | 1 | 0 | 1 | 1 | 1 |
| 79 | 1 | 1 | V6-1*02 0 / +5 / -3 D7-27*01 0 / +8 / -8 J4*02 | 1 | 1 | 0 | 1 | 1 | 1 |
| 80 | 3 | 1 | V1-46*01 0 / +6 / -8 D3-10*01 -11 / +4 / -1 J6*03 | 3 | 1 | 12 | 13 | 19 | 13 |
|  |  | 2 | V6-1*02 -6 / +9 / -5 D1-26*01 -1 / +5 / -7 J6*02 |  | 2 | 4 | 5 |  | 5 |
|  |  | 3 | V6-1*02 -6 / +10 / -6 D2-2*03 0 / +6 / -3 D3-10*01 -11 / +4 / -1 J6*03 |  | 3 | 0 | 1 |  | 1 |
| 81 | 3 | 1 | V3-33*01 -10 / +7 / 0 D6-13*01 0 / 0 / -3 J5*02 | 3 | 1 | 0 | 1 | 27 | 1 |
|  |  | 2 | V7-81*01 -3 / +18 / -4 D2-15*01 -1 / +2 / -1 J6*02 |  | 2 | 0 | 1 |  | 1 |
|  |  | 3 | V4-55*05 -6 / +3 / -3 D4-17*01 0 / +2 / 0 J5*02 |  | 3 | 24 | 25 |  | 25 |
| 82 | 3 | 1 | V2-70*11 -5 / +4 / -3 D2-8*01 -5 / +3 / -10 D3-22*01 0 / 0 / -2 J6*04 | 3 | 1 | 28 | 29 | 55 | 29 |
|  |  | 2 | V1-69*06 -23 / +14 / -7 D2-2*03 -6 / +9 / -15 J6*03 |  | 2 | 6 | 7 |  | 7 |
|  |  | 3 | V3-71*01 0 / 0 / -2 D2-2*02 -1 / 0 / -1 J6*03 |  | 3 | 18 | 19 |  | 19 |
| 83 | 1 | 1 | V1-2*02 -3 / +38 / -8 J4*02 | 1 | 1 | 0 | 1 | 1 | 1 |
| 84 | 5 | 1 | V3-13*01 -1 / +55 / -14 D2/OR15-2B*01 -7 / 0 / 0 J4*02 | 4 | 2 | 0 | 1 | 323 | 159 |
|  |  | 2 | V6-1*02 0 / +48 / -14 D2/OR15-2B*01 -7 / 0 / 0 J4*02 |  | 2 | 157 | 158 |  | 1 |
|  |  | 3 | V3-15*02 -3 / +12 / -3 D2-2*03 -5 / +9 / 0 J6*02 |  | 3 | 0 | 1 |  | 6 |
|  |  | 4 | V6-1*02 -1 / +5 / -11 D2-2*02 -1 / +9 / -9 J6*02 |  | 4 | 5 | 6 |  | 157 |
|  |  | 5 | V6-1*02 0 / +25 / -13 D3-22*01 -5 / +13 / -14 J5*02 |  | 5 | 156 | 157 |  |  |
| 85 | 1 | 1 | V1-69*09 0 / +16 / -4 J6*02 | 1 | 1 | 64 | 65 | 65 | 65 |
| 86 | 1 | 1 | V3-21*02 0 / +8 / -6 D5-12*01 -2 / +1 / -5 J1*01 | 1 | 1 | 0 | 1 | 1 | 1 |
| 87 | 2 | 1 | V3-7*01 -7 / +3 / -8 J4*02 | 2 | 1 | 130 | 131 | 133 | 131 |
|  |  | 2 | V3-13*01 -29 / +6 / -14 J6*02 |  | 2 | 1 | 2 |  | 2 |
| 88 | 3 | 1 | V1-2*02 -22 / +8 / -6 D6-13*01 -3 / 0 / -8 J4*02 | 3 | 1 | 2 | 3 | 8 | 3 |
|  |  | 2 | V3-11*01 -1 / +8 / -6 D3-10*01 -13 / +6 / -8 J6*02 |  | 2 | 3 | 4 |  | 4 |
|  |  | 3 | V1-17*02 0 / +7 / -25 D2-2*03 -20 / 0 / -8 J4*02 |  | 3 | 0 | 1 |  | 1 |
| 89 | 2 | 1 | V3-65*01 0 / +11 / -3 D3-9*01 -8 / 0 / -6 J4*02 | 1 | 1 | 126 | 127 | 128 | 128 |
|  |  | 2 | V3-23*04 0 / +22 / -4 D3-9*01 -8 / 0 / -6 J4*02 |  | 1 | 0 | 1 |  |  |
| 90 | 2 | 1 | V3-11*01 0 / +6 / 0 D3-16*02 -22 / +13 / -3 J4*02 | 2 | 1 | 1 | 2 | 7 | 2 |
|  |  | 2 | V3-49*03 -5 / +8 / -7 D2-15*01 -2 / +4 / -5 J6*02 |  | 2 | 4 | 5 |  | 5 |
| 91 | 2 | 1 | V3-13*01 0 / +29 / -7 J4*02 | 2 | 1 | 2 | 3 | 5 | 3 |
|  |  | 2 | V6-1*02 -5 / +24 / 0 J2*01 |  | 2 | 1 | 2 |  | 2 |
| 92 | 3 | 1 | V3-13*01 -10 / +1 / -2 D2-2*02 -1 / +20 / 0 J4*02 | 3 | 1 | 0 | 1 | 5 | 1 |
|  |  | 2 | V1-3*02 -1 / +9 / -3 D2-2*02 -1 / +20 / 0 J4*02 |  | 2 | 1 | 2 |  | 2 |
|  |  | 3 | V6-1*02 0 / +9 / 0 D1-1*01 -4 / +8 / -5 J6*02 |  | 3 | 1 | 2 |  | 2 |
| 93 | 3 | 1 | V3-13*01 -3 / +17 / -7 D5-5*01 0 / 0 / 0 J4*02 | 2 | 1 | 68 | 69 | 97 | 69 |
|  |  | 2 | V3-73*01 -9 / +98 / -1 D3-3*01 -5 / +24 / -5 J4*02 |  | 3 | 19 | 20 |  | 28 |
|  |  | 3 | V1-69*13 0 / +109 / -1 D3-3*01 -5 / +24 / -5 J4*02 |  | 3 | 7 | 8 |  |  |
| 94 | 2 | 1 | V1-46*03 -28 / +9 / -10 D7-27*01 -10 / +6 / -7 J4*02 | 2 | 1 | 0 | 1 | 3 | 1 |
|  |  | 2 | V4-31*01 -10 / +10 / -3 D3-22*01 -10 / +10 / 0 J5*02 |  | 2 | 1 | 2 |  | 2 |
| 95 | 3 | 1 | V3-13*01 -1 / +5 / -11 D2-15*01 -1 / +2 / -3 J6*02 | 3 | 1 | 4 | 5 | 17 | 5 |
|  |  | 2 | V4-39*01 -3 / +20 / -5 D5-12*01 -5 / +3 / -4 J4*02 |  | 2 | 0 | 1 |  | 1 |
|  |  | 3 | V1-46*01 -1 / +27 / -5 D5-12*01 -5 / +3 / -4 J4*02 |  | 3 | 10 | 11 |  | 11 |
| 96 | 3 | 1 | V3-30-3*01 -1 / +2 / -4 D6-13*01 -2 / 0 / -5 J4*02 | 3 | 1 | 17 | 18 | 37 | 18 |
|  |  | 2 | V3-21*02 0 / +7 / -9 D3-10*01 -3 / 0 / 0 J3*02 |  | 2 | 10 | 11 |  | 11 |
|  |  | 3 | V1-3*01 0 / +13 / -5 J4*02 |  | 3 | 7 | 8 |  | 8 |
| 97 | 2 | 1 | V3-30*18 -1 / +3 / -4 D3-22*01 -10 / +7 / -1 J4*02 | 2 | 1 | 71 | 72 | 82 | 72 |
|  |  | 2 | V4-61*01 -1 / +20 / -3 D5-5*01 -2 / +1 / -6 J4*02 |  | 2 | 9 | 10 |  | 10 |
| 98 | 2 | 1 | V3-73*02 -1 / +14 / -8 J4*02 | 2 | 1 | 2 | 3 | 82 | 3 |
|  |  | 2 | V3-13*01 -2 / +17 / -4 J6*03 |  | 2 | 78 | 79 |  | 79 |
| 99 | 4 | 1 | V1-68*01 -13 / +19 / -2 D6-13*01 0 / +9 / -1 J6*02 | 2 | 1 | 3 | 4 | 220 | 4 |
|  |  | 2 | V3-66*04 -4 / +6 / -4 J5*02 |  | 3 | 8 | 9 |  | 216 |
|  |  | 3 | V1-24*01 0 / +24 / -2 D4-23*01 -6 / 0 / -4 J5*02 |  | 3 | 108 | 109 |  |  |
|  |  | 4 | V4-61*01 -1 / +8 / -8 D1-7*01 0 / +1 / -4 J5*02 |  | 3 | 97 | 98 |  |  |
| 100 | 1 | 1 | V3-15*02 -5 / +12 / -5 J5*02 | 1 | 1 | 0 | 1 | 1 | 1 |
| 101 | 2 | 1 | V3-9*01 -16 / +1 / -17 D6-13*01 -12 / +4 / -6 J4*02 | 2 | 1 | 0 | 1 | 14 | 1 |
|  |  | 2 | V2-5*01 -2 / +9 / -16 D2-2*03 -3 / +5 / -4 J1*01 |  | 2 | 12 | 13 |  | 13 |
| 102 | 1 | 1 | V2-70*11 -5 / +13 / -25 D2-2*03 -22 / 0 / -4 J6*03 | 1 | 1 | 0 | 1 | 1 | 1 |
| 103 | 2 | 1 | V3-30*18 -1 / +7 / -13 D2-15*01 -2 / 0 / -1 J4*02 | 2 | 1 | 1 | 2 | 4 | 2 |
|  |  | 2 | V3-71*01 -1 / +6 / -1 J4*02 |  | 2 | 1 | 2 |  | 2 |
| 104 | 3 | 1 | V4-39*05 -1 / +7 / -8 J4*02 | 3 | 1 | 2 | 3 | 5 | 3 |
|  |  | 2 | V4-34*02 0 / +4 / -8 J4*02 |  | 2 | 0 | 1 |  | 1 |
|  |  | 3 | V4-39*01 -3 / +7 / -8 J4*02 |  | 3 | 0 | 1 |  | 1 |
| 105 | 3 | 1 | V3-66*03 -10 / +4 / -5 D1-7*01 0 / 0 / -3 J5*02 | 3 | 1 | 8 | 9 | 134 | 9 |
|  |  | 2 | V3-64*02 0 / +4 / -6 D1-7*01 0 / 0 / -3 J5*02 |  | 2 | 33 | 34 |  | 34 |
|  |  | 3 | V3-21*02 -1 / +8 / -1 D6-6*01 -4 / 0 / -3 J3*02 |  | 3 | 90 | 91 |  | 91 |
